# Data on photovoltaic power forecasting models for Mediterranean climate

**DOI:** 10.1016/j.dib.2016.04.063

**Published:** 2016-05-04

**Authors:** M. Malvoni, M.G. De Giorgi, P.M. Congedo

**Affiliations:** Department of Engineering for Innovation, University of Salento, via per Arnesano I-73100, Italy

**Keywords:** Photovoltaic Power Forecast, Least Square Support Vector Machine (LS-SVM), Group Method of Data Handling (GMDH), Multi-step ahead forecast, Forecasting errors, GLSSVM

## Abstract

The weather data have a relevant impact on the photovoltaic (PV) power forecast, furthermore the PV power prediction methods need the historical data as input. The data presented in this article concern measured values of ambient temperature, module temperature, solar radiation in a Mediterranean climate. Hourly samples of the PV output power of 960kW_P_ system located in Southern Italy were supplied for more 500 days.

The data sets, given in Supplementary material File 1, were used in DOI: 10.1016/j.enconman.2015.04.078, M.G. De Giorgi, P.M. Congedo, M. Malvoni, D. Laforgia (2015) [1] to compare Artificial Neural Networks and Least Square Support Vector Machines. It was found that LS-SVM with Wavelet Decomposition (WD) outperforms ANN method. In DOI: 10.1016/j.energy.2016.04.020, M.G. De Giorgi, P.M. Congedo, M. Malvoni (2016) [2] the same data were used for comparing different strategies for multi-step ahead forecast based on the hybrid Group Method of Data Handling networks and Least Square Support Vector Machine. The predicted PV power values by three models were reported in Supplementary material File 2.

**Specifications Table**

**Table:** 

Subject area	Engineering, Neurosciences, Physics
More specific subject area	Renewable resource, Solar energy, Photovoltaic, Forecasting methods
Type of data	Table, Excel files
How data was acquired	Pyranometer and temperature probe allow measurement of the weather data. A data logger integrated to inverter DC/AC returns the PV output power. The SCADA system archives the historical data that are available in reserve access web site.
Data format	Analyzed, processed
Experimental factors	The samples are treated to align them at the same time step.
Experimental features	Historical weather data and measured PV output power to train the forecasting models.Predicted PV power by LS-SVM, GMDH and GLSSVM models with Direct strategy at five time horizons.
Data source location	Lecce, Italy (40° 19׳32"׳16 N, 18° 5׳52"׳44 E)
Data accessibility	Data are within this article

## 1 Value of the data

• Weather and power historical data can be used to forecast the PV power at short or long time horizons applying different forecasting models.
• The forecasted PV power by LS-SVM, GMDH and GLSSVM can be used in future works to compare the performance of different prediction models.
• The PV power prediction data can be used in power system models to simulate the planning and dispatching operations of the electric grid.
• PV power forecasting represents a valid support in the electric energy market analysis.

## 2 Data

The data refer to a PV system located in Mediterranean Climate. They consist of the meteorological data as hourly mean ambient temperature (*T_a_*), hourly mean module temperature (*T_m_*), hourly mean solar irradiance measured on two tilted planes (*I*_3_ and *I*_15_) and the hourly mean PV power for a period of 21 months. The forecasted hourly PV power values, as the output of three prediction models at 1, 3, 6, 12 and 24 h ahead, are provided.

## 3 Experimental design, materials and methods

### 3.1 Description of PV system and data acquisition

The collected data are related to the 960kW_P_ photovoltaic system, which is located in the campus of the University of Salento, in Monteroni di Lecce (LE), Puglia (40° 19׳32"׳16 N, 18° 5׳52"׳44 E). The PV modules were installed on shelters used as car parking. The PV plant is divided by two sub-fields (Table 1) that have the same azimuth (10°) and different tilt of modules (3° and 15°). More details of the technical features are reported in [3].

Suitable instruments were installed to monitor main weather parameters. Pyranometers LP PYRA 02 provide for the measurement of solar radiation on two different module׳s planes with a typical sensitivity of 10 W/m^2^. PT100 temperature sensors measure the PV module temperature and the ambient temperature. A supervisory system, integrated into the inverter DC/AC supplies PV output power. A SCADA system “SIMATIC WinCC” provides to archive measured data that are available on the ESAPRO private website [4].

A reserved access website allows to download following collected data in a specified time period:

• *T_s_* ambient temperature and module temperature measured every 10 min (°C);
• *I_s_* solar irradiance on the tilted plain with angle of 3° and 15° sampled every 1 min (W/m^2^);
• *P_s_* photovoltaic output power measured every 1 min (W).

### 3.2 Experimental data pre-processing

The weather data have a relevant impact on the PV power forecast [5], [6]. In order to take into account the influence of the meteorological parameters on the forecasting of the PV power, the weather data are used as input for the forecasting models. For this aim, it needs to refer the measured weather data to the same sample steps, chosen equal to 1 h in this work. Therefore, hourly mean values of temperature and solar irradiance are defined as follows:

• *T_a_*(*i*) and *T_m_*(*i*) are the hourly average values of the ambient temperature and the module temperature in the previous 60 min with respect to the hour *i*
  (1) $$T(i)=\frac{1}{6}\sum_{t=1}^{6}T_{s}(t)$$
• *I*_3_(*i*) and *I*_15_(*i*) are the hourly average values of the solar irradiance on module plain, inclined of angle 3° and 15° respectively in the previous 60 min with respect to the hour *i*

(2) $$I(i)=\frac{1}{60}\sum_{t=1}^{60}I_{s}(t)$$

In addition, we defined *P*(*i*) as the hourly average value of the PV output power, produced in the previous 60 min *t* with respect to the hour *i,* given by:

(3) $$P(i)=\frac{1}{60}\sum_{t=1}^{60}P_{s}(t)$$

The data supplied in this work are related to the historical measurements from 05/03/2012 to 31/12/2013. The collected records, defined as in (1), (2), (3), are reported in supplementary material, File 1 that contains 15.828 sample rows.

In order to apply the forecasting methods, we define the target $\hat{P_{h}}\left(i\right)$ as the sum of *h* hourly power consecutive values respect to the hour *i*, given by:

(4) $$\hat{P_{h}}(i)=\sum_{i+1}^{i+h}P\left(k\right)$$

where *h* is the time horizon from 1 to 24 and *i* represents the hourly time instant. The target data $\hat{P_{h}}$ given by Eq.(4) are provided in Supplementary material, File 2.

Data processing

The weather data and the target data $\hat{P_{h}}$, as previously described, represent the input for three forecasting models: Least Square Support Vector Machine (LS-SVM), Group Method of Data Handling (GMDH) and hybrid algorithm (GLSSVM) [2]. Each model has implemented to predict the PV power at 1, 3, 6, 12 and 24 h ahead.

The MATLAB software has been used to design and simulate three forecasting models under Intel(R) Xeon(R) CPU E5-1650 3.20 GHz CPU and 8-GB RAM. The 65% of total records are used to train the models and the 35% remaining of them (5.467) to the testing. Supplementary material, File 2 reports the predicted PV power as output of three models with direct strategy for five time horizons. More details are given in [2].

## Figures and Tables

**Table 1:** Technical specifications of the PV sub-fields.

**Subfield**	
*PV1*	
Nominal power of PV system	353.3 kW_p_
Azimuth	−10°
Tilt	3°
Total number of modules	1104
Net modules׳ surface	1733.3 m^2^
*PV2*	
Nominal power of PV system	606.7 kW_p_
Azimuth	−10°
Tilt	15°
Total number of modules	1896
Net modules׳ surface	2976.7 m^2^

## Footnotes

Appendix A Supplementary data associated with this article can be found in the online version at doi:10.1016/j.dib.2016.04.063.
